# Unintentional Infusion of Insulin Into the Epidural Space During Labor

**DOI:** 10.7759/cureus.65371

**Published:** 2024-07-25

**Authors:** Sara S Neves, Joana Almeida

**Affiliations:** 1 Department of Anesthesiology, Centro Hospitalar Universitário de Santo António, Porto, PRT

**Keywords:** obstetrics, medication errors, labor, epidural analgesia, drug administration routes

## Abstract

Inadvertent injection of drugs into the epidural space has a potential for serious morbidity and is probably underestimated and underreported.

A 39-year-old female with no medical history presented for delivery. An epidural catheter was requested and correctly placed. Continuous epidural infusion was chosen for labor analgesia. Six hours after the parturient complained about inefficient analgesia, a syringe swap with insulin was identified.

Despite the risk of possibly neurotoxic preservatives in the insulin formulation, no neurological sequelae were observed.

This case highlights the issue of wrong-route drug administration and the urgent need to adopt route-specific connections.

## Introduction

“To err is human,” said Alexander Pope. Nevertheless, err should involve discussing the process that led to its occurrence. Reporting these incidents should aim to raise awareness of its potential. Medication errors are far from rare. Inadvertent epidural administration of drugs not approved for this route increases the risk of morbidities, such as paraplegia or even death [1].

Syringe exchanges and wrong-route drug administration, such as epidural/intravenous administration, are two of the most common errors. Although syringe swaps mainly involve injecting intravenous agents, which may or may not be labeled, a direct connection of an intravenous line to the epidural filter with a Luer lock coupling may also occur [1].

This study presents a case of an accidental insulin infusion by an epidural catheter during vaginal labor.

## Case presentation

A 39-year-old pregnant female, primigravida and 40 weeks of gestation, presented for delivery induction with no relevant personal history. Throughout the first stage of labor, the parturient requested an epidural catheter for analgesia. The neuraxial technique was performed with the patient in the left lateral decubitus position and after local anesthetic injection. An 18-G epidural needle was inserted in the L4-L5 interspace in the midline, and three cm of the catheter was introduced cephalic in the epidural space. A mixture of 10 mcg of sufentanil and 10 mL of 0.2% ropivacaine was slowly administrated, over 10 minutes, while carefully monitoring the parturient and fetus. Patient-controlled epidural analgesia (PCEA) was set up with a background adjustable infusion of 0.1% ropivacaine and 0.25 mcg/mL sufentanil at 8 mL/h and an 8 mL bolus with a 30-minute lockout.

Nearly six hours later, the patient complained about inefficient analgesia that was successfully relieved with 8 mL of 2% lidocaine. During the active phase of the first stage of labor, approximately two hours after the previous evaluation, the pain resurfaced. The nurse in charge noticed an erroneous insulin (Actrapid®, 1 U/mL) syringe infusing through the epidural catheter. There was a type-I diabetic parturient in the same obstetric ward requiring an insulin infusion.

The on-call anesthesiologist was notified, and the patient was immediately assessed following a systematic Airway, Breathing, Circulation, Disability, and Exposure (ABCDE) approach. Hypoglycemia (56 mg/dL) was detected and corrected with an oral intake of 15 g of fast-acting carbohydrate. In total, 25 units of short-acting insulin were injected into the epidural space. No hemodynamic instability was observed. A summary neurological examination revealed no deficits. The parturient vital signs, neurological status, along fetal HR were monitored continuously until delivery.

After administering saline solution (5 mL), 8 mL of 0,2% ropivacaine was injected and PCEA restarted with pain relief. The anesthesiologist confirmed the correct IV insulin infusion in the type-I diabetic parturient, discharging the hypothesis of syringe swap.

Due to prolonged labor, an urgent cesarean section was performed 24 hours after the event, converting epidural analgesia to epidural anesthesia without intercurrences. The postoperative course was unremarkable, with efficient postoperative epidural analgesia.

A physical examination was performed at three-time points: 24 hours, 48 hours, and hospital discharge. No neurological deficits were observed. The patient was informed of the warning signs motivating a reassessment in the emergency department.

## Discussion

The incidence of erroneous epidural administration is probably underestimated [2]. A large variety of drugs are inadvertently applied epidurally. Fortunately, in most reported cases, little harm has resulted [1,3]. Some complications have been reported, with the most severe cases being attributed to the epidural administration of potassium chloride solutions. These incidents resulted in a variety of symptoms, including pain and persistent paraplegia [1,4].

This case report described the accidental epidural administration of 25 units of insulin during labor analgesia. There was a type-I diabetic parturient with an insulin infusion in the same ward. The tactical preparation of syringes with a mixture of 0.1% ropivacaine with 0.025 mcg of sufentanil in the night shift alongside the storage of these syringes in the anesthesia cart imposes a risk of syringe swap.

In a 2014 National Patient Safety Agency report on safer administration of insulin, cases resulting in severe harm and death from dosing errors in insulin administration were highlighted [5].

Continuous intravenous insulin infusion is a safe and effective method for glucose control during the peripartum period in diabetic parturients [6]. The effect of epidural insulin infusion on blood glucose levels remains uncertain. However, the epidural space is well vascularized, which might lead us to think insulin would rapidly be absorbed into the circulation [1]. In this case, a singular asymptomatic episode of hypoglycemia was reported and promptly corrected. Despite the risk of potentially neurotoxic additives in the insulin formulation, the patient had no neurological sequelae. Therefore, we postulated that the prior administration of approximately 50 mL of local anesthetic through the epidural catheter may have diluted the accidentally administered epidural insulin concentration, potentially lowering the risk of injury.

Safety measures and protocols are being developed to prevent erroneous drug administration related to drug preparation, administration, and labeling [1,7]. It has been recommended that medications and syringes designated for epidural use should be stored separately from those intended for intravenous use. Likewise, intravenous and epidural infusion pumps should be physically separated. In this case, the error resulted from an unusual set of circumstances in which an insulin syringe intended for a different patient was inadvertently placed in the epidural anesthesia cart, along with several epidural analgesia syringes. Clear labeling of epidural catheters, epidural infusion syringes, and pumps contributes to the reduction of medication errors [4,8]. Nevertheless, as in this present case, most errors occur with syringes that are labeled correctly [9].

Human awareness is a crucial factor in reducing the number of unintentional drug administrations. Ideally, different connection latches should be used for intravenous and epidural lines as this would guarantee that there could be no accidental misconnections, thus ensuring their proper linkage [1,10,11]. In this case, the hospital promptly received notification regarding the accidental administration, leading to the introduction of new syringes with specific connections for epidural use to minimize the risk of similar errors.

Medical errors can have profound implications, including patient harm, prolonged hospitalization, increased healthcare costs, and adding further strain on healthcare resources and providers. In alignment with international patient safety goals set by the World Health Organization (WHO), preventing medication administration errors is crucial for safeguarding patient health and ensuring high-quality healthcare worldwide [12]. Therefore, our priority should be continuous training, skill and quality improvement, learning from errors, and education about the importance of delivering safe and effective care [13].

Considering that reporting errors is fundamental to their prevention, this report aims to implement improvements that enhance the safety of both patients and healthcare professionals [14].

## Conclusions

Medication errors, while inevitable to some extent, can be significantly reduced through targeted interventions. This case emphasizes the importance of continuing staff education, inventory management protocols, and the careful design of medical equipment. Specifically, designing epidural infusion systems that are incompatible with intravenous systems and establishing distinct storage areas for different medication categories are crucial steps in preventing such errors.

Effectively mitigating medication errors requires a comprehensive examination of the underlying processes that contribute to their occurrence. Reporting such incidents should not only raise awareness of potential consequences but also drive meaningful improvements in safety protocols and practices.
